# “Fit for the finals” – project report on a telemedical training with simulated patients, peers, and assessors for the licensing exam

**DOI:** 10.3205/zma001599

**Published:** 2023-04-17

**Authors:** Sigrid Harendza, Lisa Bußenius, Julia Gärtner, Miriam Heuser, Jonathan Ahles, Sarah Prediger

**Affiliations:** 1 Universitätsklinikum Hamburg-Eppendorf, III. Medizinische Klinik, Hamburg, Germany; 2 Albert-Ludwigs-Universität Freiburg, Medizinische Fakultät, Studiendekanat, Freiburg, Germany

**Keywords:** exam, formative testing, simulation, telemedicine, training

## Abstract

**Background:**

Undergraduate medical students take the licensing exam (M3) as a two-day oral-practical examination. The main requirements are to demonstrate history taking skills and coherent case presentations. The aim of this project was to establish a training in which students can test their communication skills during history taking and their clinical reasoning skills in focused case presentations.

**Methods:**

In the newly developed training, final-year students took four telemedical histories in the role of physicians from simulated patients (SP). They received further findings for two SPs and presented these in a handover, in which they also received a handover of two SPs which they had not seen themselves. Each student presented one of the two received SPs in a case discussion with a senior physician. Feedback was given to the participants on their communication and interpersonal skills by the SPs with the ComCare questionnaire and on the case presentation by the senior physician. Sixty-two students from the universities of Hamburg and Freiburg in their final year participated in September 2022 and evaluated the training.

**Results:**

Participants felt that the training was very appropriate for exam preparation. The SPs' feedback on communication and the senior physician's feedback on clinical reasoning skills received the highest ratings in importance to the students. Participants highly valued the practice opportunity for structured history taking and case presentation and would like to have more such opportunities in the curriculum.

**Conclusion:**

Essential elements of the medical licensing exam can be represented, including feedback, in this telemedical training and it can be offered independent of location.

## Zusammenfassung

**Hintergrund:**

Den dritten Abschnitt der Ärztlichen Prüfung (M3) absolvieren Medizinstudierende als zweitägige mündlich-praktische Prüfung. Es sind vor allem Anamnesefähigkeiten und schlüssige Fallpräsentationen zu demonstrieren. Ziel dieses Projektes war es, ein Training zu etablieren, in dem Studierende ihre kommunikativen Fähigkeiten in Anamnesegesprächen und ihre Clinical-Reasoning-Fähigkeiten in fokussierten Fallvorstellungen erproben können.

**Methoden:**

Im neu entwickelten Training führten PJ-Studierende in der Rolle von Ärzt*innen vier telemedizinische Anamnesegespräche mit Simulationspatient*innen (SP). Zu zwei SPs erhielten sie weitere Befunde und stellten diese in einer Fallübergabe vor, in der sie zwei SPs übergeben bekamen, die sie selbst nicht gesehen hatten. Eine*n der beiden gehörten SPs stellte jede*r in einer Fallbesprechung mit einer Oberärztin vor. Feedback erhielten die Teilnehmenden von den SPs zu ihren kommunikativen und interpersonellen Fähigkeiten mit dem ComCare-Bogen und von der Oberärztin zur Fallpräsentation. Es nahmen 62 PJ-Studierende der Universitäten Hamburg und Freiburg im September 2022 teil und evaluierten das Training.

**Ergebnisse:**

Die Teilnehmenden hielten das Training zur Examensvorbereitung für sehr geeignet. Das Feedback der SPs zur Kommunikation und der Oberärztin zu den Clinical-Reasoning-Fähigkeiten erhielt in der Wichtigkeit für die Studierenden die höchsten Bewertungen. Die Teilnehmenden schätzten die Übungsmöglichkeit für strukturierte Anamneseerhebung und Fallpräsentation sehr und wünschten sich mehr solcher Angebote im Studium.

**Schlussfolgerung:**

Im telemedizinischen Training lassen sich wesentliche Elemente des Dritten Abschnitts der Ärztlichen Prüfung mit Feedback abbilden und es kann standortunabhängig angeboten werden.

## 1. Introduction

Regardless of their completion in a regular or a model curriculum, undergraduate medical studies in Germany end, according to the licensing regulations (ÄApprO), with the final licensing exam (M3) (§ 30 ÄApprO, see [http://www.gesetze-im-internet.de/_appro_2002/BJNR240500002.html]). This is an oral-practical exam that takes place on two days. The first day of the exam is reserved for the practical examination with patient presentation (§ 30 para. 1 ÄApprO). In the M3 exam, the examinee has to show that s*he knows how to apply the knowledge acquired during his or her studies in practice, that s*he has the necessary skills and abilities to conduct medical conversations (§ 30 Abs. 3 ÄApprO) and that s*he knows how to behave in accordance with the general rules of medical behavior towards the patient (§ 30 Abs. 3 S. 10 ÄApprO). In particular, the examinee should demonstrate that s*he has mastered the technique of taking a medical history and of basic laboratory methods and is able to assess their results, is able to obtain and request the required information to make a diagnosis, to recognize the different significance and weighting for making a diagnosis and to critically utilize it within the frame of differential diagnostic considerations (§ 30 para. 3 p. 2-3 ÄApprO). This process is referred to as clinical reasoning [1], [2], [3] and represents the basis of medical thinking and action. The assessors for the M3 exam are appointed by the state examination office of the respective federal state on the recommendation of the universities and are usually at least board certified medical specialists. Their tasks include ensuring that the exam is conducted in accordance with the ÄApprO, grading the exam, and documenting the content of it. 

During undergraduate medical training, the opportunity to prepare for this type of oral-practical exam is very limited, as the majority of exams during the semesters are multiple-choice or objective structured clinical examinations (OSCEs), even in relatively newly established universities [4]. Also, there are only a few published teaching formats in German-language medical curricula that support the explicit learning and practice of clinical reasoning [5], [6], [7], [8], [9], [10], [11], [12], [13], although the introduction of clinical reasoning in medical curricula is explicitly demanded in the European higher education area [14] and a standard work – meanwhile in its second edition [15] – as well as further didactic instructions for teaching clinical reasoning have been available internationally since 1991 [16], [17]. An explanatory model for clinical reasoning as well as for many other decision processes is the so-called dual-process theory [18], [19]. While the intuitive way of thinking is applied, for example, in multiple-choice questions and is, therefore, learned implicitly [20], the analytical way of thinking, if not explicitly taught, can be observed, for example, through the behavior of physician role models in case discussions when they justify their working hypotheses and further diagnostic or therapeutic steps. That these two ways of thinking are used intermittently in everyday medical practice and typical cognitive errors occur in both, intuitive and analytical thinking, has also been studied [21], [22]. The use of clinical reasoning can be assessed during history taking [23], [24] as well as during case presentations [5], [25].

Some medical faculties and also commercial companies or medical professional associations offer seminars to prepare for the licensing exam in order to familiarize oneself with the specific circumstances of the examination situation. However, there is usually no particular focus on physician-patient communication and clinical reasoning. However, physician-patient communication is an essential component in the parts of the M3 exam that take place with patients, and clinical reasoning is a crucial prerequisite for focused case presentations and discussions, which is an important part of the M3 exam, both at the bedside and without patient participation in additional cases. Therefore, the aim of this project was to develop a training that students can undergo towards the end of their final year in order to test their communication skills in while taking focused histories and their clinical reasoning skills for the focused presentation of patients as well as to receive feedback on this. This is intended to enable final-year students to prepare for the oral-practical exam in a way that is more tailored to their needs and oriented on the expression of their own competences in these two areas.

## 2. Project description

**Figure 1:**
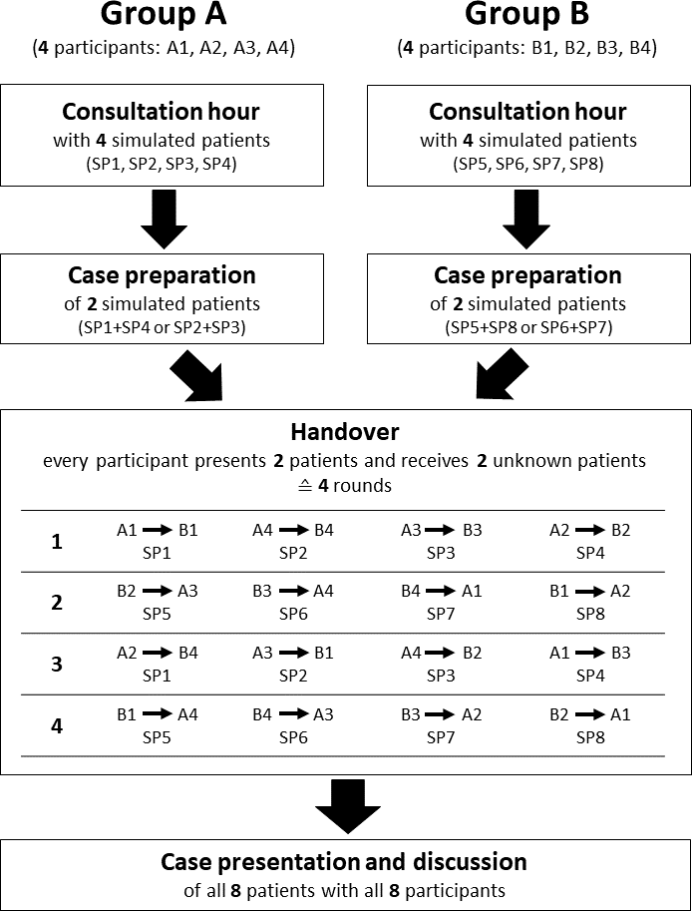
Procedure of the telemedical training “Fit for the finals”

In 2020, we developed a competence-based telemedicine training for undergraduate medical students in their final year at the Center for the Development and Assessment of Medical Competences at the University Medical Center Hamburg-Eppendorf [26]. This training included a telemedical consultation with four simulated patients per participant, patient documentation and ordering of further diagnostics using an electronic patient file, as well as a case presentation per participant in a digital case discussion with a senior physician. It represents a development towards telemedicine of two previous projects, where we developed and validated a training format for a simulated first day of work as a physician based on essential facets of competence for physicians at the beginning of their postgraduate training [27], [28], [29], [30]. The previously established telemedicine training format [26] was redesigned for the “Fit for the finals” training as follows (see figure 1 (Fig. 1)).

**Figure 2:**
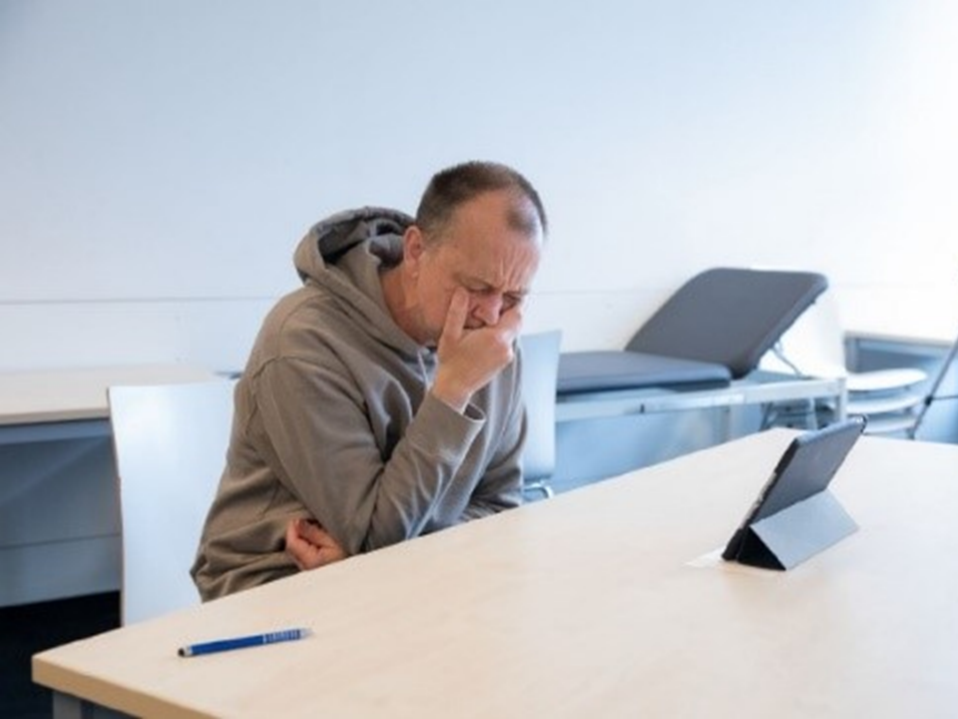
Simulated patient in telemedicine setting with tablet

All participants had received a written briefing for the training on the content and technical procedure in advance, including further documents from the UKE clinical reasoning course [5] for focused history taking and case presentation with reasoning. The main aspects were repeated in a personal briefing by the organizer of the training and the participants had the opportunity to ask questions. Analogous to the previous telemedicine-based training [26], a telemedical consultation hour with four simulated patients per participant took place in the first phase (consultation hour). Eight students per round participated in the training at the same time (group A and group B), whereby the patient cases for group A and B were different. Figure 2 (Fig. 2) shows a simulated patient in the telemedicine setting with tablet; a total of eight tablets were required. All patient cases were designed according to real patients from the emergency department of the University Medical Center Hamburg-Eppendorf and included internal and surgical diseases that are frequently assessed in the final exam. Furthermore, in addition to a chief complaint, all patient cases were again designed with a personal situation that presented a communicative challenge [26]. The roles were played by professional actors and actresses who were specially selected for the respective roles and trained by SH and SP for the physician-patient interviews and the completion of the evaluation forms (see attachment 1 ). Each interview was scheduled for a maximum of 10 minutes. All interviews were recorded on video. The participants were provided with the corresponding findings of the physical examination after each encounter with the simulated patients, and during the five minutes until the next interview the participants could think about the previous case including the physical finding. The simulated patients electronically completed the ComCare questionnaire after each interview, a validated instrument for measuring communicative and interpersonal skills [31], [32], which contains open and closed questions. Eight tablets were also required for this purpose. The participants received the results of these questionnaires with the quantitative evaluation of the items as well as their personal feedback after the end of the training.

**Figure 3:**
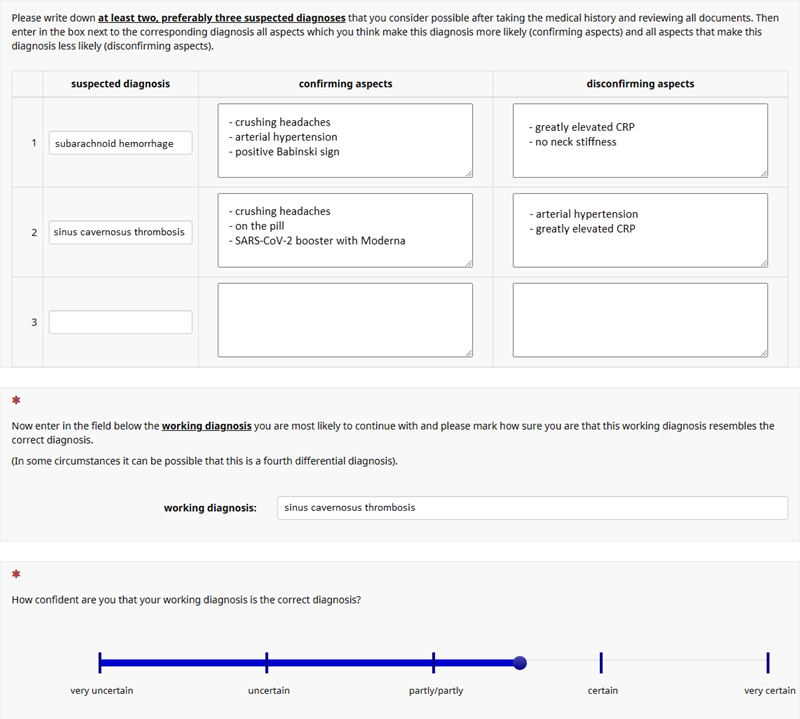
Digital form for the preparation of the case presentation

In a second phase (case preparation) after the telemedical consultation hour, the participants received further findings for two of their four patients, e.g. laboratory results, ECG, X-rays or other findings. They were also given an electronic form for each of the two patients in which they should document several differential diagnoses to structure the case presentation. For each differential diagnosis, participants were asked to document in two boxes (“confirming aspects” and “disconfirming aspects”) the information that made the respective differential diagnosis more or less likely based on the patient's history, the physical examination, and the additional diagnostic findings. This electronic form was modeled after a virtual patient program for clinical reasoning training [33]. Finally, participants were asked to document the working diagnosis they wanted to hand the two cases over with. They were also asked to use a slider (from “very uncertain” to “very certain”) to indicate how certain they felt with each working diagnosis after weighting the differential diagnoses by arguments (see figure 3 (Fig. 3)).

In the third phase (handover), the participants of group A reported to the participants of group B in different rounds on one of their two patients and vice versa, whereby for each conversation the participants were virtually shifted in such a way that they met new dialog partners each time. Four laptops were required for this purpose. With this approach, the respective receiving person took on the role of an assessor in these peer case handovers. Their task was to understand the received case and to discuss it with the person presenting it, in order to be able to present one of the received cases later in a structured way by themselves. This procedure was intended to simulate the situation of an actual handover and thus, at the same time, to focus attention on the essential aspects of a case. In the briefing text, all participants had received an example of a focused case presentation showing how to use the confirming and disconfirming aspects from the electronic form for clinical reasoning in weighing differential diagnoses. Six minutes were available for each handover. The case handovers were also video recorded.

In the fourth phase (case presentation and discussion), all eight participants of groups A and B met digitally with a senior physician via laptop. The participants were informed beforehand which of the two received patients they had to present. The patients were called up individually and the participants then had ten minutes to introduce each patient, discuss them with the senior physician and the peers, look at essential findings (e.g. ECGs or X-rays) together, and medically solve the cases with regard to further needed diagnostics and therapy. In addition, the participants received feedback on their clinical reasoning from the senior physician. Finally, a debriefing of the training took place with the eight participants of each round as a group discussion. These two phases were also videotaped. The discussion was transcribed verbatim for the evaluation of the students' contributions.

In September 2022, a total of 62 students (47 from the University of Hamburg and 15 from the University of Freiburg) participated in the “Fit for the finals” training over two days shortly before completing their final year. Their mean age was 27.6±3.7 years. Of the 62 participants, 80.6% were female, 19.4% male. Students had been informed via digital bulletin boards or email of the opportunity to participate in this voluntary training, and registration was on a first-come, first-served basis. For logistical reasons, the invitation to the Hamburg students was issued two weeks earlier than the invitation to the Freiburg students. For the scientific monitoring of this project, an approval of the Ethics Committee of the Hamburg Chamber of Physicians was obtained (reference number: PV3649) and the students consented to participate in writing. The participants received a digital questionnaire to evaluate the training after the debriefing in which they answered questions about their own experiences during the training, about the training as a whole as well as about its phases and the organization of the training on a 5-point Likert scale (1: does not apply, 2: does rather not apply, 3: partly/partly, 4: rather applies, 5: fully applies).

## 3. Results

In the assessment of their communicative and interpersonal skills by the simulated patients with the ComCare, the students received on average 4.15±0.45 points of a maximum of 5 points (see table 1 (Tab. 1)). In particular, “use of comprehensible language” (4.71±0.32), “comprehensible explanation of next diagnostic and therapeutic steps” (4.41±0.41), and “attentive listening” (4.29±0.52) were rated highest by the simulated patients. The item “the physician was interested in me as a person and in my environment” received the lowest rating of 3.45±0.67 from the perspective of the simulated patients.

After experiencing the training situation, the students rated themselves most confident in dealing with the patients (4.08±0.86) and least confident in clinical reasoning (3.31±0.88) (see table 2 (Tab. 2)). They considered the patient cases very useful for practicing differential diagnostic thinking and the interviews with the simulated patients for practicing focused history taking (4.85±0.41 and 4.76±0.50, respectively). Feedback from the simulated patients on their own communication skills and feedback from the teacher on the presentation of a patient case were very important to participants (4.79±0.49 and 4.90±0.31, respectively). In the free text comments of the evaluation, the constructive feedback of the simulated patients and the teacher as well as the variety and depth of the real patient cases were also mentioned to be essential aspects of the training. In addition, the open learning atmosphere and the role change into the position of an assessor (as a receiver of a handover) were found to be helpful. The debriefing groups revealed that the participants had recognized essential principles of clinical reasoning for themselves: “[...] as long as you can justify your decisions [...] everything is okay”, “[...] you don't need to stressed out, if you don't know something, just explain your ideas” and “if [your concept] is not conclusive, you [have] to question it”. In addition, it turned out that for many participants there had apparently been little opportunity to practice structured case presentations during the final year or there were also concerns about not receiving adequate feedback (“unfortunately, I never had my own patients [to] deliver a structured presentation”; “I missed real professional exchange and confident answers from senior physicians very much”; “[...] depending on the person leading the ward rounds, you might think twice [whether to present a patient or not], because it might sometimes be difficult with some personalities”).

Overall, the participants rated the “Fit for the finals” training with a school grade of 1.2±0.41. They were very satisfied with the organizational communication and processes (4.76±0.50), as well as with the technical processes of the training (4.25±0.88). They considered the training to be very suitable as preparation for the M3 exam (4.56±0.65) and would recommend the course to their fellow students (4.88±0.33). Reasons given for recommending it to others included “[...] because you gain confidence and realize that you don't have to know everything”; “[...] because you take on the role of the receiver and then the presenter”; “[...] because mistakes are not seen as a problem but as an opportunity to learn the systematic approach”.

## 4. Discussion and conclusion

The evaluations of the simulated patients and the feedback of the students show that the two main goals of the training, to provide the participants with feedback on their communication competence during history taking and on their clinical reasoning competence, were achieved. The redesigned training for the M3 exam allowed participants to test their own communication and clinical reasoning skills, so that they think, with the appropriate feedback they can adapt the preparations for their oral-practical exam to their needs. As the results of the ComCare questionnaire show, the students achieved good ratings in the communicative aspects, while there is still room for improvement in some interpersonal aspects. The participants of the training found the additional feedback from the simulated patients very helpful. This is consistent with findings that feedback from simulated patients helps to improve students' communication skills [34]. The participants of the training also experienced the interaction with the simulated patients themselves as useful for their own learning due to the authenticity of the cases. That interactions with simulated patients also contribute to professional development already during the interaction was shown in another study [35].

By changing roles and thus perspectives from presenting to receiving and back to presenting a case again, participants reported experiencing essential aspects of clinical reasoning in discussion with their peers in terms of focusing and reasoning as is required when presenting patient cases in the oral-practical exam. A meta-analysis on feedback students received on their clinical performance during examinations showed highly variable and, in some cases, poorly beneficial results with regard to the usefulness of this feedback for their own learning and personal development [36]. Peer feedback within the case discussion phase of our training was found to be very useful in improving one's case presentation skills, especially due to the change in roles. The teacher's feedback on the patient presentation and clinical reasoning process was also very important to the participants for their own learning, as there had apparently been little opportunity for many participants to practice clinical reasoning and case presentations with feedback during their studies. With appropriate teacher training on clinical reasoning [37], it should be relatively easy, with reasonable effort, to provide students with learning opportunities on clinical reasoning and case presentation in other phases of their studies, so that they could use a training such as the one in this project even more effectively for self-assessment of their skills. However, various aspects have been identified that stand in the way of implementing a longitudinal clinical reasoning curriculum [38] and need to be considered individually at different study locations in order to successfully implement clinical reasoning. Should the implementation of a clinical reasoning curriculum prove difficult, at least regular feedback from teachers or even peers seems to be helpful for learning communication and other clinical skills [39], [40], [41].

Even though only a small sample of 62 voluntary students from two medical schools participated in the training in a first run, it could already be shown that the intended learning objectives were achieved from the participants' point of view. It can be assumed that these results can also be transferred to a larger sample. By its format and with appropriate feedback, the training helps students to reflect on their personal skills with regard to communication and clinically well-argued case presentation, to identify possible deficits, and thus, from their perspective, to better set their own priorities in preparation for the oral-practical exam. As other elements of the training that have not been used so far, an individual analysis of the history taking and case discussion videos with individual feedback by lecturers or peers would be possible. Due to the telemedical training approach, the training can be very easily offered nationwide and independent of location, as demonstrated in this study.

## Funding

This project was supported by the Joachim Herz Foundation.

## Ethics

This project was conducted in accordance with the Declaration of Helsinki and the Ethics Committee of the Hamburg Chamber of Physicians approved the study and confirmed its innocuousness. The project included a written consent of the participants for study participation including digital recording and a retention of all collected records for at least ten years and the participation was voluntary and anonymized (reference number: PV3649). 

## Acknowledgement

We thank the medical students of the Universities of Hamburg and Freiburg who participated in the training and the actresses and actors Theresa Berlage, Jantje Billker, Christian Bruhn, Claudia Claus, Christiane Filla, Uwe Job, Thomas Klees, Frank Thomé. Many thanks for the photograph (figure 2) to Axel Kirchhof.

## Competing interests

The authors declare that they have no competing interests. 

## Supplementary Material

Roles of the simulated patients

## Figures and Tables

**Table 1:**
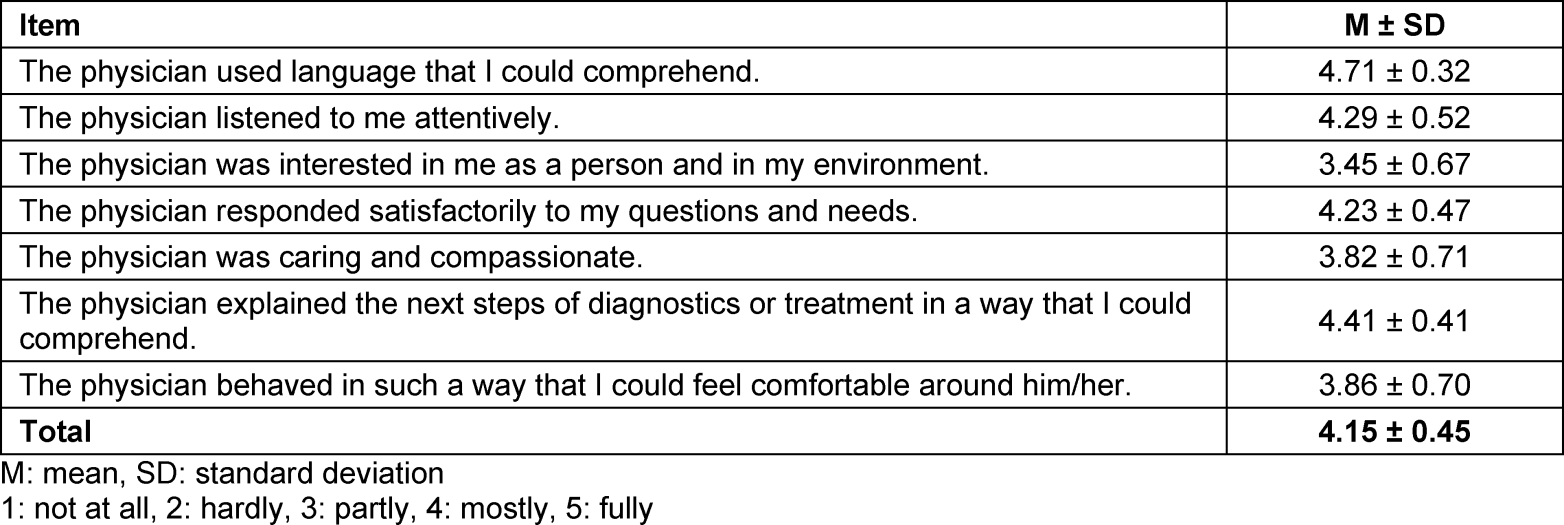
ComCare results of the participants

**Table 2:**
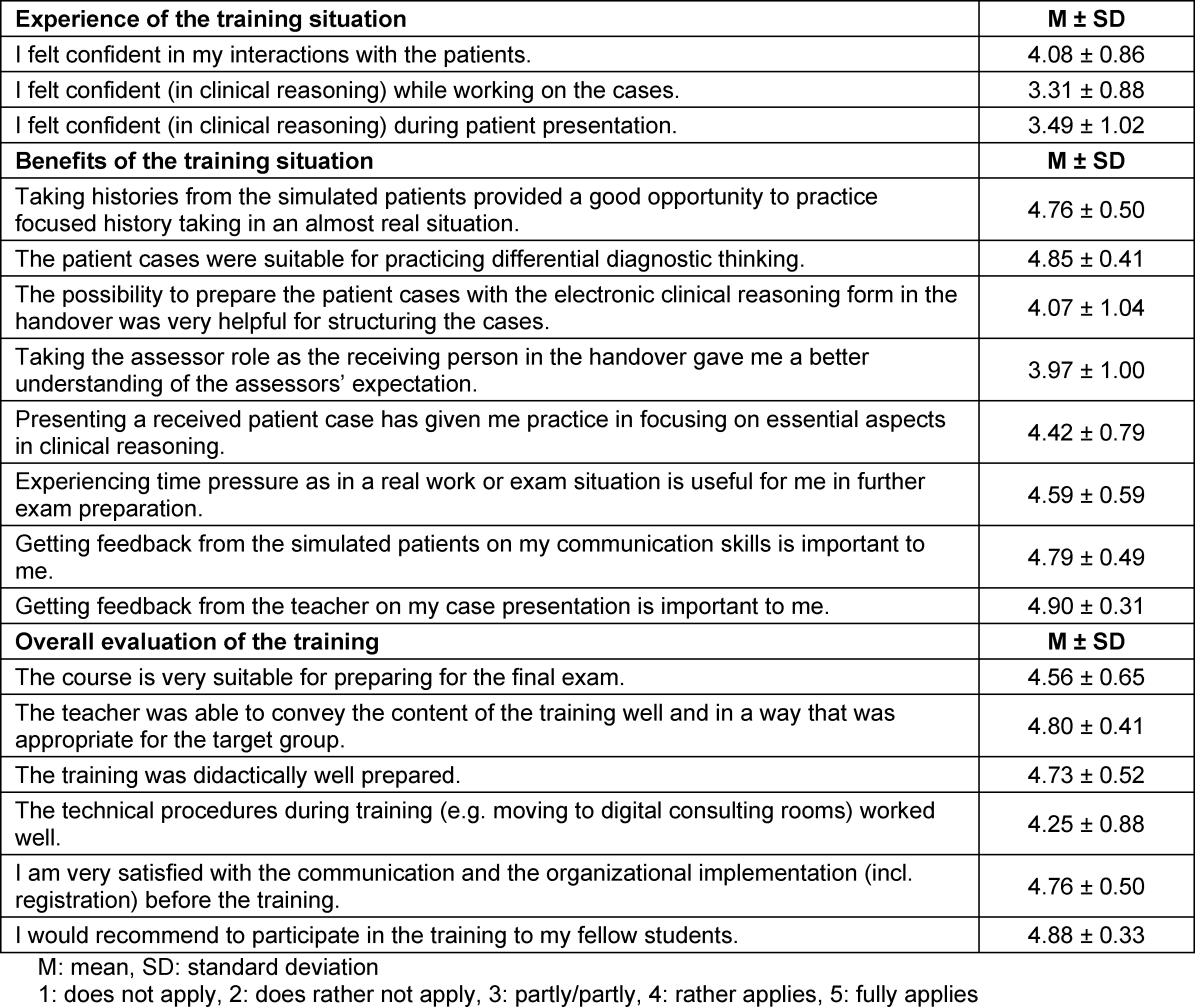
Evaluation of the "Fit for the finals" training by the participants
