# Supplementary material for: “Fit for the finals” – project report on a telemedical training with simulated patients, peers, and assessors for the licensing exam
Source: GMS J Med Educ. 2023 Apr 17;40(2):Doc17. doi: 10.3205/zma001599 (PMC10285374; doi:10.3205/zma001599)
Supplement: Roles of the simulated patients [file JME-40-17-s-001.pdf]

Attachment 1: Roles of the simulated patients

SP	Sex	Age	Chief complaint	Communicative challenge	Diagnosis
A1	M	56	progressive fatigue and hemoptysis	downplaying of symptoms ("exaggerated", "only stress-related")	granulomatous polyangiitis (GPA)
A2	F	45	severe headache	annoyed; concerned about possible side effect of Moderna vaccination	community-acquired pneumonia
A3	M	54	very severe abdominal pain	homeless; alcoholic; mother tongue not German	perforated pyloric ulcer
A4	F	40	unwanted weight loss	exhausted; very worried about having cancer	endocarditis
B1	F	47	fatigue	esoterically interested; skeptical about taking medication	chronic renal failure
B2	M	62	joint pain and numbness	somewhat concerned; answers questions very specifically	multiple myeloma
B3	F	61	left lower abdominal pain	friendly, but taciturn because of the severe pain	covered perforated sigmoid diverticulitis
B4	M	56	severe neck pain	pragmatic; hard-headed; concealing	posterior myocardial infarction (NSTEMI)

SP: Simulated patient, M: male, F: female

Attachment 1 to Harendza S, Bußenius L, Gärtner J, Heuser M, Ahles J, Prediger S. *“Fit fort he finals” – project report on a telemedical training with simulated patients, peers, and assessors fort he third part oft he medical lecensing exam*. GMS J Med Educ. 2023;40(2):Doc17. DOI: 10.3205/zma001599
